# Streptococcus mutans and Streptococcus sobrinus detection by Polymerase Chain Reaction and their relation to dental caries in 12 and 15 year-old schoolchildren in Valencia (Spain)

**DOI:** 10.4317/medoral.18941

**Published:** 2013-05-31

**Authors:** Mateo Sánchez-Acedo, José M. Montiel-Company, Francisco Dasí-Fernández, José M. Almerich-Silla

**Affiliations:** 1 Departament of Stomatology, University of Valencia, Spain; 2 Departament of Stomatology, University of Valencia, Spain; 3 Fundación Investigación Hospital Clínico Universitario de Valencia/INCLIVA. Valencia, Spain; 4 Departament of Stomatology, University of Valencia, Spain

## Abstract

A cross-sectional study was carried out to determine the prevalence of Streptococcus mutans and Streptococcus sobrinus and the association of the two in a random sample (n=614) of the child population of the region of Valencia (Spain). Saliva samples were analyzed by the quantitative polymerase chain reaction (PCR) method to study the relation of these bacteria to caries prevalence and the DMFT index. The prevalence of S. mutans was 35.4% at age 12 and 22.9% at age 15, that of S. sobrinus 18.9% and 8.4% and that of the S. mutans-S. sobrinus association 18.2% and 6.8% respectively. At both 12 and 15 years of age, the caries prevalence rates were lower in the Streptococcus-free group of children (37.6% and 48.5% respectively) and higher in the S.mutans-only group (67.3% and 74.0%). At the age of 12, the DMFT index was significantly higher in the mutans-only carriers (2.1) than in the Streptococcus-free and S. mutans-S. sobrinus association groups (both 0.9). At the age of 15, the DMFT index was significantly higher in the S. mutans-S. sobrinus association (3.71) and mutans-only (3.1) carrier groups than in the Streptococcus-free group (1.4). Determination of S. mutans and S. sobrinus by real-time quantitative PCR can provide valuable information for caries risk assessment in epidemiological studies.

** Key words:**Streptococcus mutans, Streptococcus sobrinus, polymerase chain reaction, dental caries, cross-sectional studies.

## Introduction

Dental caries presents a multifactorial aetiology, as environmental factors, food and hygiene habits and individual genetic susceptibility all play an important role in the development of this disease (1).

Streptococcus mutans and *Streptococcus sobrinus* are strongly implicated in the onset of human dental caries. Numerous studies have shown the association of *S. mutans* and *S. sobrinus* with higher caries levels (2-4) and several studies have found that *S. sobrinus* prevalence is more strongly associated with high caries activity than that of *S. mutans* (5-6). It is considered that determination of these two species in early childhood could be important for diagnosing and preventing dental caries.

Some authors (7-8) have developed PCR methods to detect *S. mutans* in samples of dental plaque. To facilitate biological sample collection for epidemiological studies, it has been developed a PCR method to detect *S. mutans* and *S. sobrinus* in saliva, using specific primers for the genes that encode glucosyltransferase (gtfB in *S. mutans* and gtfI in *S. sobrinus*), which can be used to assess the prevalence of these organisms in epidemiological studies (9).

Based on a strong correlation between microorganism counts in saliva and plaque, *S. mutans* determination in saliva has been suggested as a suitable method for identifying patients at high risk of dental caries (10). As saliva is continuously in contact with all the teeth, it provides a better reflection of the colonization of mutans streptococci on all dentition (11).

The objectives of this study were to determine the prevalence of *S. mutans* and *S. sobrinus* and of the association of *S. mutans* and *S. sobrinus* in the 12 and 15 year-old child population of the region of Valencia (Spain), employing a quantitative PCR method in saliva samples, and to examine the relation of these bacteria to caries prevalence and the DMFT index.

## Material and Methods

-Study group 

A cross-sectional epidemiological study was conducted in a random sample of a child population of 12 and 15 years of age in the Valencia region of Spain. The child population of the Valencia region comprises 45,000 children aged 12 years and a similar number aged 15 years, who attend more than 1,500 primary and secondary schools in the region. Random sampling of clusters (classes in schools) was conducted, resulting in the selection of classes in 27 schools for observations of 12-year-old children and classes in 20 schools for observations of 15-year-old children (12).

Of the population examined, saliva samples were only collected from children whose parents signed an informed consent form, resulting in a final sample size for this study of 303 children out of the 478 examined at the age of 12 and 311 out of the 401 examined at the age of 15 years.

-Clinical examination

To ensure the reliability of the measurements, caries diagnosis calibration of the 6 dentists recruited for the study was carried out during the weeks prior to commencement of the study. The three dentists with the highest agreement, assessed using the Kappa index with a gold standard-experienced examiner, were appointed as examiners. The Kappa values for the gold standard examiner comparisons were 0.91, 0.86, and 0.85, respectively, for the three examiners.

The clinical examinations were performed in the schools, with the child sitting on a chair, using a portable 60 W white–blue spectrum lamp as the source of illumination. No more than 25 children were examined during a session to avoid the effects of visual tiredness.

The examination instruments employed were a WHO type periodontal probe and a no. 5 plain mouth mirror. Each examination team was provided with 35 sets of sterilized probes and mirrors, each in a sealed bag, placed in a portable plastic container. The field work was carried out during November and December 2004.

-Written questionnaire

To collect information on oral health-related behaviours, the children were asked to complete a written questionnaire. The questionnaire consisted of multiple choice test questions which had already been used and validated in other studies in Spain (12). Included in the questions were requests for information on tooth brushing frequency and on the frequency of intake of cariogenic foods. Fluoride rinses carried out once a week at school was also recorded.

-Measurements

The WHO caries criteria (13) were employed for diagnosing and coding all the tooth examined. Two outcome variables were considered: the DMFT count and caries prevalence (DMFT > 0) at the age of 12 and 15.

The fulfillment of the school-based fluoride mouth-rinsing programs was recorded as high if weekly school-based fluoride rinsing had been carried out for at least one academic year prior to the examinations. Otherwise it was considered low.

The intake of cariogenic foods was considered high if cariogenic foods (foods with a high refined sugar content) were eaten between meals every day or almost every day. Otherwise the cariogenic food intake was considered low.

Finally, the daily tooth brushing habit was recorded as appropriate if the child reported brushing his or her teeth at least once a day, otherwise the daily tooth brushing habit was considered inappropriate.

-Saliva Samples

The saliva was collected with a no.50 sterile paper point, held in forceps with sterile beaks and placed on the tip of the child’s tongue for 1 minute until soaked with saliva. Each point was then transferred with the forceps to a sterile Eppendorf tube. The tubes were transported in a portable refrigerator to a freezer where they were stored at -80°C. None of the journey times exceeded 3-4 hours.

-DNA isolation of S. mutans and S. sobrinus from saliva samples

The isolation and purification of the genomic DNA was carried out with the ChargeSwitch Forensic DNA Purification kit (Invitrogen). (Invitrogen Corporation 1600 Faraday Avenue Carlsbad, California 92008).

-Quantitative PCR 

Calculation of copy number

Standard curves were obtained using the bacterial strains *S. mutans* (CCUG 11877T, serotype c; Clarke 1924-AL) and *S. sobrinus* (CCUG 27507, serotype d; Coykendall 1983-VP). The bacterial strains were cultured on blood agar plates and then subjected to 0.5 McFarland suspension (equivalent to 1.5 x 108 CFU/ml). Serial dilutions were performed to obtain bacterial suspensions from 1 x 100 to 1 x 107 CFU. Quantitative detection of *S. mutans* and *S. sobrinus* was performed using the TaqMan assay (14). Briefly, gene-specific primer pairs (200 nmM each) and probes (250 nM) for *S. mutans* and *S. sobrinus* were used with 1X TaqMan Universal PCR Master Mix (Applied Biosystems) and 5 µl of isolated bacterial DNA in 20 µl reaction volume. The PCR conditions were 10 min at 95ºC for enzyme activation, followed by 45 two-step cycles (15 sec 95ºC; 1 min at 58ºC). Detection was performed using the ABI PRISM 7900 Sequence Detection System. Standard curves for each organism were obtained from the amplification of genomic DNA from samples containing 1.0 x 100 to 1.0 x 107 CFU. Each sample was analyzed in triplicate and the Ct value of each sample was converted to quantity of *S. mutans* and *S. sobrinus* using the standard curves measured in the same experiment. The linearity and sensitivity of the assay was also determined from these standard curves by qPCR.

The detection and quantification was linear over a range from 1.0 x 102 and 1.0 x 107 CFU per reaction mixture for both *S. mutans* and *S. sobrinus*. This assay was used to determine the numbers of *S. mutans* and *S. sobrinus* from 303 children aged 12 and 311 aged 15 years.

-Statistical analysis

The data were analysed with the SPSS 19.0 statistics application. The samples were divided into four Streptococcus carrier status groups: free of Streptococci, carrying only *S. mutans*, carrying only *S. sobrinus*, or carrying an association of *S. mutans-S. sobrinus*. A univariate analysis was performed to compare the mean DMFT values using the ANOVA test or Student’s t test. A chi-square test was used for testing differences in caries prevalence. A logistic regression analysis was also performed with caries prevalence as the dependent variable and free of Streptococci, carrying only *S. mutans*, carrying only *S. sobrinus*, carrying an association of *S. mutans-S. sobrinus*, daily tooth brushing, intake of cariogenic food, fluoride rinsing and age as independent variables.

## Results

In the 12-year-old group the mean DMFT was 1.13 (95% confidence interval CI-95% 0.94-1.32) and the caries prevalence was 52.2%. At the age of 15, the results were DMFT 1.81 (CI-95%: 1.53-2.009) and caries prevalence 54.3%.

The *Streptococcus mutans, S. sobrinus* and *S. mutans-S. sobrinus* association prevalence data are shown in  Table 1. The prevalence of *S. mutans* was 35.4% at age 12 and 22.9% at age 15, that of *S. sobrinus* 18.9% and 8.4% and that of the *S. mutans-S. sobrinus* association 18.2% and 6.8% respectively.

**Table 1:**
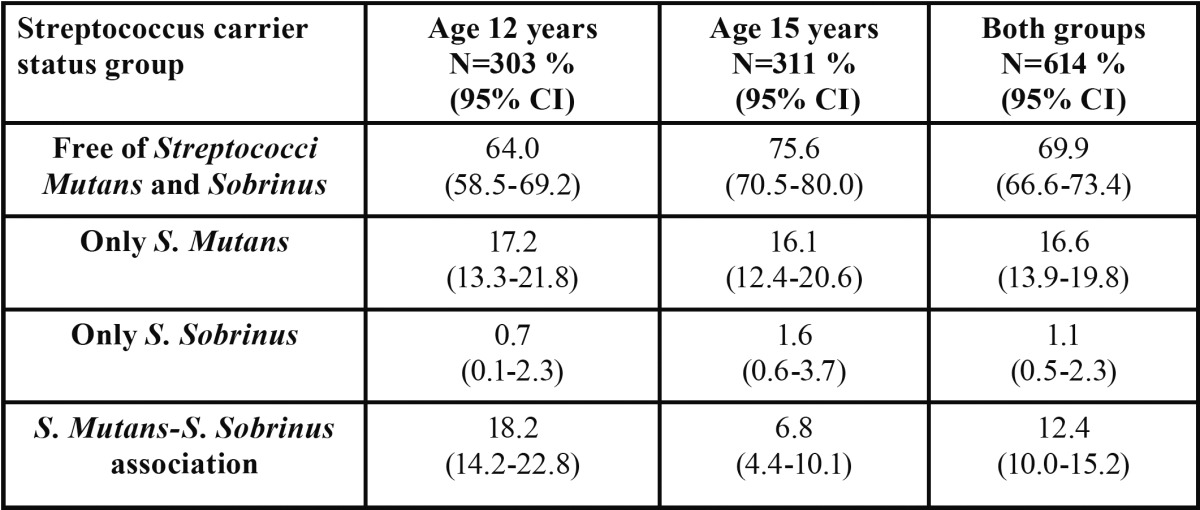
Prevalence of Streptococci Mutans and Sobrinus.

The mean *S. mutans* level was 6.47x104 CFU/ml (1.81x103-1.28x105) at 12 years of age (n=303) and 2.11x104 (5.95x103-3.63x104) at 15 years (n=311). The *S. sobrinus* levels were 1.44x104 CFU/ml (3.04x103-2.56x104) and 1.36x103 CFU/ml (0-2x103) respectively.

The different groups exhibit significant differences in caries prevalence and DMFT ( Table 2). At both 12 and 15 years of age, the *Streptococcus*-free group of children had lower caries rates than the other groups and the *S.mutans*-only group presented the highest caries prevalence. Among the 12 year-olds, the DMFT score was significantly higher in the mutans-only group than in the *Streptococcus*-free and *S. mutans-S. sobrinus* association groups. Among the 15 year-olds, the DMFT score was significantly higher in the *S. mutans-S. sobrinus* association and mutans-only groups than in the *Streptococcus*-free group. In the sample as a whole, the caries prevalence and DMFT index were significantly higher in the mutans-only carriers than in the *Streptococcus*-free and *S. mutans-S. sobrinus* association groups.

**Table 2:**
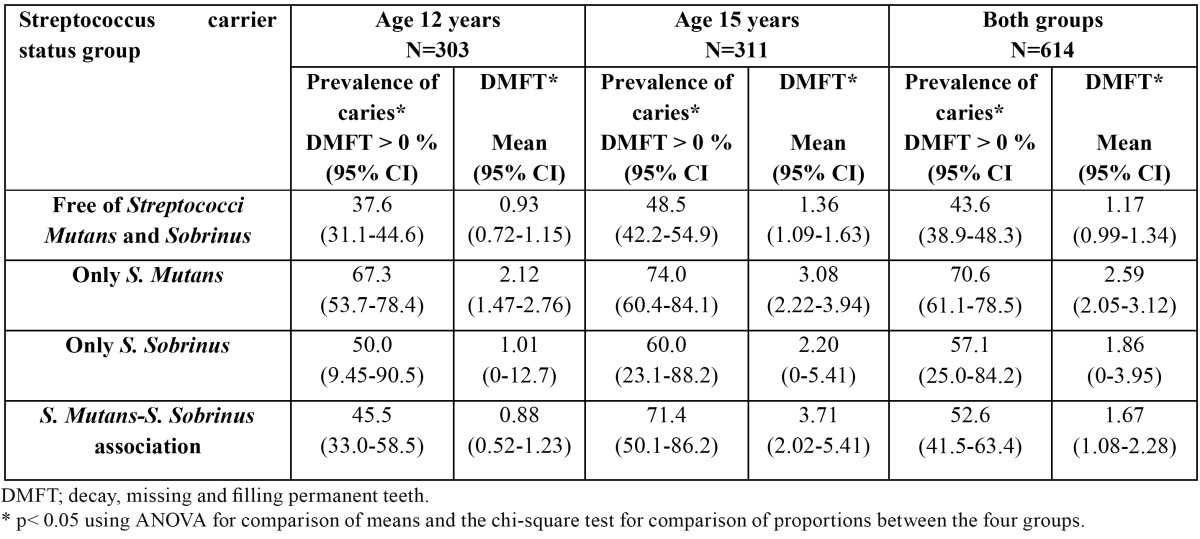
Streptococcus carrier status group in relation to caries prevalence and the DMFT index.

In  Table 3 *Streptococcus*-free group was higher and *S. mutans* group lower in DMFT index scores? 3 than scores >3 at both 12 and 15 year-olds. Significant differences were exhibit between the four groups by DMFT index scores in both.

**Table 3:**
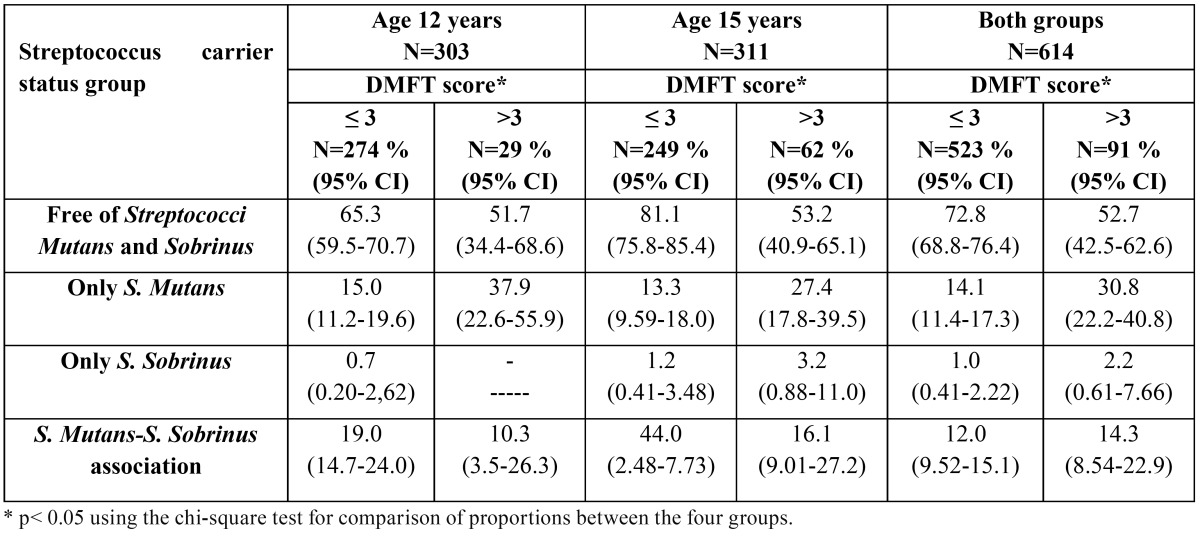
Relation between DMFT score (? 3 or >3) and *Streptococcus carrier status group*.

 Table 4 shows the results of the multiple logistic regression analysis with caries prevalence as the dependent variable. Carrying only *S. mutans*, daily intake of cariogenic foods and age were significantly associated with caries prevalence in this sample.

**Table 4:**
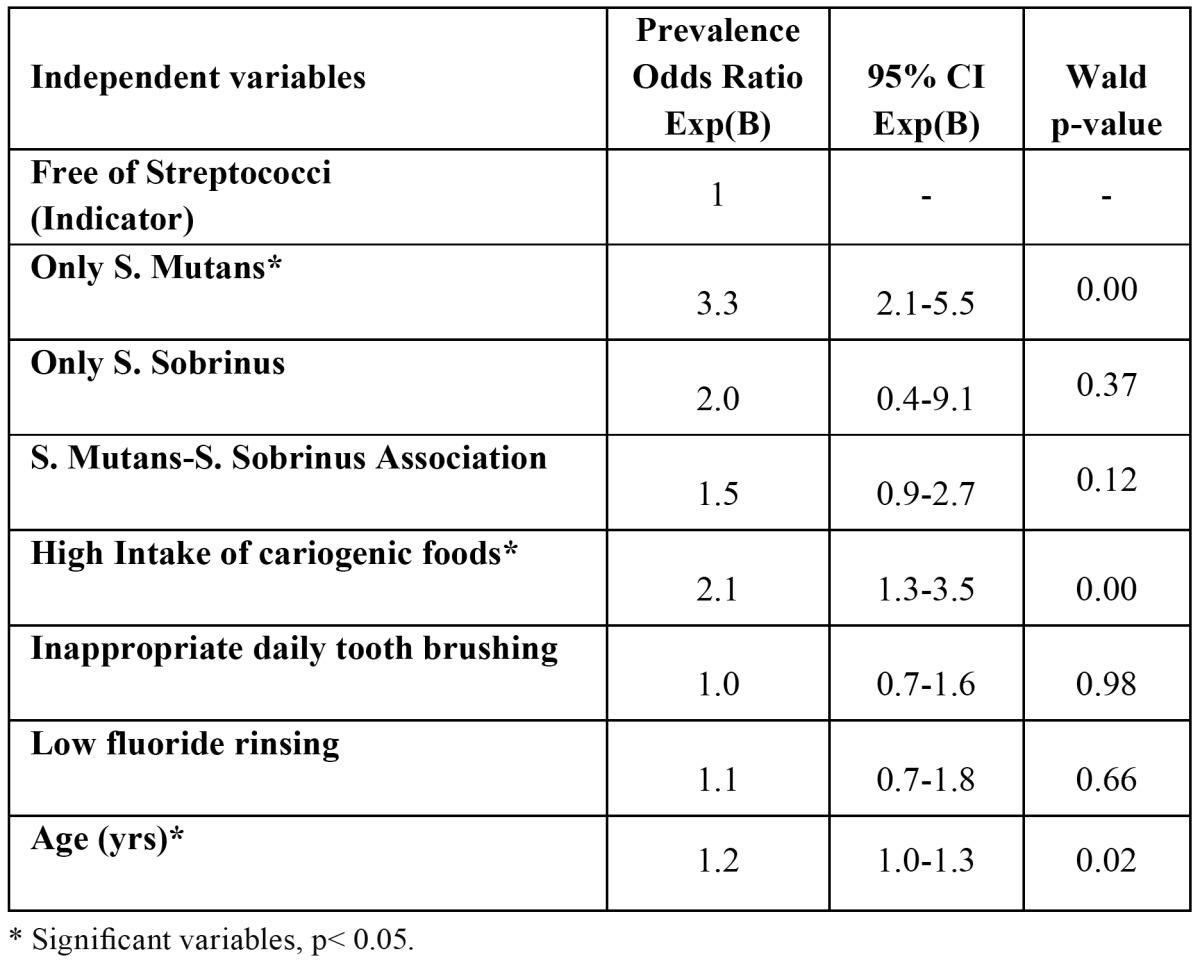
Multiple logistic regression analysis with caries prevalence (DMFT >0) as the dependent variable (n=614).

## Discussion

The aetiology of caries is multifactorial in character, comprising genetic, behavioural, environmental and microbial factors (15). Caries is a polymicrobial infection (16). Every bacterial species plays a role in determining the cariogenicity of the biofilm or dental plaque. In this study we analyzed samples of unstimulated saliva to determine the presence of *S. mutans* and *S. sobrinus* and to relate it to the presence or absence of caries in schoolchildren in the Valencian Community.

The system we employed to detect *S. mutans* in samples of unstimulated saliva by means of a polymerase chain reaction (qRT-PCR) has been used in other studies and has shown itself to be valid and reliable (14), although the limitations of cross-sectional studies for establishing associations between microorganisms and dental caries should not be forgotten (17).

Determination of *S. mutans* and *S. sobrinus* CFU/ml levels by real-time quantitative PCR has been shown to be a reliable, fast method that is applicable in epidemiological studies (4,9).

S. mutans and *S. sobrinus* are the species that have been most frequently isolated from the human oral cavity and have been implicated as the main germs causing dental caries in humans (18).

Numerous studies have shown that the presence of *S. sobrinus* is associated with greater caries activity (19). *S. mutans* is the predominant species and is sometimes the only one isolated. In the present study, as in other (20), when *S. sobrinus* was isolated *S. mutans* was almost always found to be present as well.

The results of this study show *S. mutans* prevalences of 36% at age 12 and 24.5% at age 15, *S. sobrinus* prevalences of 18.9% and 8.4% and *S. mutans-S. sobrinus* association prevalences of 18.2% and 6.8% respectively. These figures are well below those obtained in other studies, which range from 51% to 100% for *S. mutans* and from 23% to 83% for *S. sobrinus* (2-3,21-23).

There are several possible explanations for our low prevalence of *Streptococcus mutans* and *S. sobrinus*. Firstly, the published epidemiological studies with determinations in saliva of *S. mutans* and *S. sobrinus* by qPCR technologies, are based on a low number of samples, ranging from 80 (4) to 140 samples (11), compared to 614 samples in the present study.

Another important point is that the child population of the region of Valencia, where the random representative sample was obtained, is characterized by low levels of caries, access to public fissure sealant programs, school-based fluoride mouth-rinsing and a high percentage of tooth brushing, which could influence the low prevalence.

A last point that should be considered when evaluating the data is that our study was based on captured rather than stimulated saliva, and it could be that stimulating the saliva with paraffin, a procedure used in other studies, disturbs the bacterial biofilm that adheres to the enamel surface, leading to a greater number of bacteria being found in the saliva. Although collecting the saliva samples from the tongue might bias the results, there are studies that have found a relationship between the quantities of *S. mutans* in plaque and saliva, so if a large number of germs are found in the saliva, the number in the plaque will also be high (24-25). As regards the relationship between *S. mutans* and dental caries, we found significantly higher caries prevalence in the *S. mutans* carriers among both groups of children. We also found a positive relationship between *S. mutans* prevalence and the DMFT index, as did other (26).

With regard to the *S. mutans- S. sobrinus* association, in the present study the 15-year-old children with this association exhibited higher DMFT counts, similar to reports in other studies which show that the association of these two bacteria is related to higher caries levels (2-4).

However, the present study did not find this association either in the 12 year-old group or on analyzing the sample as a whole. Longitudinal studies indicate that children harbouring both *S. mutans* and *S. sobrinus* have a significantly higher incidence of dental caries than those who are positive for *S. mutans* alone (3).

In order to establish the relationship between caries and the presence of certain bacteria, it should be borne in mind that caries lesions pass through various stages according to their depth and that this will influence the presence and predominance of certain microorganisms and the decrease or absence of others (27).

A systematic review of the literature (28) confirms that *S. mutans* plays an important role in the initiation of dental caries of the enamel and root. Nevertheless, some recent studies indicate that the relation between *S. mutans* and caries is not absolute: high proportions of *S. mutans* can persist on tooth surfaces without injury occurring and caries can develop in the absence of this species (29).

Caries occurs as a result of a complex interaction between dental plaque (whose physiological characteristics may be modified by carbohydrates in the diet), the diet, and oral hygiene practices. These interactions are difficult to understand, especially as our knowledge of the microflora associated with health, disease and the transition from health to disease is very limited (30). In a multiple logistic regression analysis with caries prevalence as the dependent variable, carrying *S. mutans* only, intake of cariogenic foods and age have proved to be significant independent variables.

Our results might suggest a clear association between caries and the presence of *Streptococccus mutans* but do not confirm such a relationship between caries and the *S. mutans-S. sobrinus* association. In populations with low caries levels, the prevalence of *S. mutans* and *S. sobrinus* in unstimulated saliva is not as high as might be expected. Their determination in epidemiological studies can provide valuable information for caries risk assessment, although the multifactorial nature of this disease should not be forgotten.
